# ASPC president's page: Preparing the next generation of leaders in preventive cardiology

**DOI:** 10.1016/j.ajpc.2025.101086

**Published:** 2025-08-22

**Authors:** Michael D. Shapiro

**Affiliations:** Fred M. Parrish Professor of Cardiology and Molecular Medicine Director, Center for Prevention of Cardiovascular Diease Department of Cardiovascular Medicine Wake Forest University School Medicine, Winston-Salem, NC 27157, 336-716-2718, USA

Dear ASPC Members and Colleagues,

Over the past several decades, preventive cardiology has moved from the periphery of clinical practice to its very center. For much of the twentieth century, prevention was shaped by landmark epidemiologic discoveries, including recognition of hypercholesterolemia and hypertension as modifiable risk factors and the demonstration that lifestyle change could alter cardiovascular risk. Yet despite these insights, prevention was often viewed as an adjunct to “real” cardiology, which focused on treating acute events.

The landscape today is entirely different. Preventive cardiology now commands a robust evidence base, a sophisticated set of diagnostics and therapeutics, and a central place in cardiovascular guidelines. Imaging modalities such as coronary artery calcium scoring are widely used to refine risk assessment. Lipid management has evolved from a singular focus on LDL cholesterol to a broader approach that includes apoB, Lp(a), and triglyceride-rich lipoproteins. The pharmacologic toolkit has expanded well beyond statins, with PCSK9 inhibitors, bempedoic acid, inclisiran, and investigational Lp(a)-lowering therapies changing the treatment horizon. Systemic inflammation is recognized as a modifiable driver of atherosclerosis. The scope of prevention now extends to heart failure, arrhythmias, and vascular health across the lifespan.

This transformation has been driven by committed clinicians, scientists, and public health leaders who insisted that preventing the first event should be as urgent as treating the last one. It also reflects growing recognition by health systems and policymakers that prevention is not only clinically effective but economically essential. The best heart attack remains the one that never happens.

The American Society for Preventive Cardiology (ASPC) has been a central voice in this evolution. Since its founding, the Society has provided a professional home for those dedicated to advancing prevention through science, education, and advocacy. ASPC members have led pivotal trials, authored guidelines, and driven implementation strategies that have changed practice worldwide. We have convened a multidisciplinary community that includes cardiology, endocrinology, epidemiology, primary care, nursing, pharmacy, nutrition, and exercise physiology, recognizing that prevention thrives when disciplines work together.

We are now at an inflection point. The science of prevention continues to accelerate, and the expectations for clinicians are expanding alongside it. Digital health tools, advanced imaging, and molecular diagnostics are no longer distant innovations, they are part of the contemporary preventive practice environment. The challenge is not simply keeping up with new tools, but integrating them thoughtfully, ensuring they improve outcomes and reach all patients. That integration will not happen by chance; it will be led by clinicians and scientists who have been deliberately prepared for that responsibility.

This is why preparing the next generation of leaders in prevention is one of ASPC’s highest priorities. Our aim is not only to produce well-trained clinicians, but to cultivate individuals with the vision, skills, and resilience to lead in a rapidly changing environment. They must be able to interpret evolving science, integrate new technologies into patient care, address disparities with urgency, and advocate effectively for prevention at the policy level. Leadership in preventive cardiology now requires fluency in areas ranging from data science and genomics to behavioral economics and health systems design alongside the human skills of communication, mentorship, and collaboration.

ASPC has taken concrete steps to meet this need. The **ASPC Training Academy** offers a structured educational pathway that blends foundational knowledge with emerging science and practical application. The **CardioCore** lecture series provides high quality, timely content to keep members informed and engaged across diverse practice settings. The ***Heart of Prevention*** podcast features the leading voices in the field and the new frontiers they are driving forward. Our **mentorship program** connects early career members with established leaders who can offer guidance in clinical practice, research strategy, and career development.

We are also committed to giving early career members tangible opportunities to lead. This includes **co-authoring consensus statements, chairing sessions** at our annual congress, participating in **working groups**, and representing ASPC in collaborative efforts with other societies. These experiences accelerate professional growth, expand networks, and prepare future leaders to assume greater responsibilities within ASPC and beyond.

The trajectory of the past few decades shows what is possible when prevention is prioritized. Smoking rates have declined, lipid control has improved, and survival after acute events has increased. Yet significant challenges remain. There are persistent disparities, rising obesity and diabetes rates, and the continued influence of social determinants of health. The next generation will inherit both the successes we have achieved and the unfinished work we leave behind.

Our responsibility is clear: equip them to build on progress while confronting the realities we have yet to change.

The ASPC will remain at their side. We will provide the platform, the mentorship, and the vision to ensure that the field continues to grow in depth, relevance, and impact. The future of preventive cardiology will not be defined solely by the discoveries we make, but by the leaders we prepare. That is our commitment and our legacy.

Warmest regards

## CRediT authorship contribution statement

**Michael D. Shapiro:** Conceptualization, Writing – original draft, Writing – review & editing.

## Declaration of competing interest

The authors declare the following financial interests/personal relationships which may be considered as potential competing interests: Michael D. Shapiro reports a relationship with Amgen, Arrowhead, Ionis, Novartis, New Amsterdam, Merck, Regeneron, Aidoc, Novo Nordisk, and Tourmaline. that includes: consulting or advisory. Michael D. Shapiro reports a relationship with Amgen, Arrowhead, Boehringer Ingelheim, Esperion, Novartis, Ionis, Merck, New Amsterdam, Lilly, and Cleerly that includes: funding grants. If there are other authors, they declare that they have no known competing financial interests or personal relationships that could have appeared to influence the work reported in this paper

